# Microbial vitamin biosynthesis links gut microbiota dynamics to chemotherapy toxicity

**DOI:** 10.1128/mbio.00930-25

**Published:** 2025-05-20

**Authors:** Lars E. Hillege, Kai R. Trepka, Benjamin G. H. Guthrie, Xueyan Fu, Romy Aarnoutse, Maia R. Paymar, Christine Olson, Chen Zhang, Edwin Ortega, Lorenzo Ramirez, Judith de Vos-Geelen, Liselot Valkenburg-van Iersel, Irene E. G. van Hellemond, Arnold Baars, Johanna H. M. J. Vestjens, John Penders, Adam Deutschbauer, Chloe E. Atreya, Wesley A. Kidder, Marjolein L. Smidt, Janine Ziemons, Peter J. Turnbaugh

**Affiliations:** 1 GROW–Research Institute for Oncology and Reproduction, Maastricht University 568601 , Maastricht, Limburg, the Netherlands; 2 Department of Surgery, Maastricht University Medical Center+ 199236, Maastricht, Limburg, the Netherlands; 3 Department of Microbiology and Immunology, University of California San Francisco 214560, San Francisco, California, USA; 4 USDA Human Nutrition Research Center on Aging, Tufts University 1810, Medford, Massachusetts, USA; 5 Division of Medical Oncology, Department of Internal Medicine, Maastricht University Medical Center+ 382937, Maastricht, Limburg, the Netherlands; 6 Department of Medical Oncology, Catharina Hospital 3168, Eindhoven, North Brabant, the Netherlands; 7 Department of Medical Oncology, Hospital Gelderse Vallei 3096 , Ede, Gelderland, the Netherlands; 8 Department of Internal Medicine, VieCuri Medical Centre 8187 , Venlo, Limburg, the Netherlands; 9 NUTRIM–Institute of Nutrition and Translational Research in Metabolism, Maastricht University 5211, Maastricht, Limburg, the Netherlands; 10 Department of Medical Microbiology, Infectious Diseases, and Infection Prevention, Maastricht University Medical Center+ 199236, Maastricht, Limburg, the Netherlands; 11 Environmental Genomics and Systems Biology Division, Lawrence Berkeley National Laboratory 1666, Berkeley, California, USA; 12 Department of Medicine, University of California San Francisco 166668, San Francisco, California, USA; 13 Helen Diller Family Comprehensive Cancer Center 151233, San Francisco, California, USA; 14 Chan Zuckerberg Biohub-San Francisco 578083, San Francisco, California, USA; University of Washington, Seattle, Washington, USA

**Keywords:** human gut microbiome, colorectal cancer, chemotherapy, vitamin K, metagenomics

## Abstract

Dose-limiting toxicities pose a major barrier to cancer treatment. While preclinical studies show that the gut microbiota influences and is influenced by anticancer drugs, data from patients paired with careful side effect monitoring remains limited. Here, we investigate capecitabine (CAP)-microbiome interactions through longitudinal metagenomic sequencing of stool from 56 advanced colorectal cancer patients. CAP significantly altered the gut microbiome, enriching for menaquinol (vitamin K2) biosynthesis genes. Transposon library screens, targeted gene deletions, and media supplementation revealed that menaquinol biosynthesis protects *Escherichia coli* from drug toxicity. Stool menaquinol gene and metabolite levels were associated with decreased peripheral sensory neuropathy. Machine learning models trained in this cohort predicted toxicities in an independent cohort. Taken together, these results suggest treatment-associated increases in microbial vitamin biosynthesis serve a chemoprotective role for bacterial and host cells. Further, our findings provide a foundation for in-depth mechanistic dissection, human intervention studies, and extension to other cancer treatments.

**IMPORTANCE:**

Side effects are common during the treatment of cancer. The trillions of microbes found within the human gut are sensitive to anticancer drugs, but the effects of treatment-induced shifts in gut microbes for side effects remain poorly understood. We profiled gut microbes in colorectal cancer patients treated with capecitabine and carefully monitored side effects. We observed a marked expansion in genes for producing vitamin K2 (menaquinone). Vitamin K2 rescued gut bacterial growth and was associated with decreased side effects in patients. We then used information about gut microbes to develop a predictive model of drug toxicity that was validated in an independent cohort. These results suggest that treatment-associated increases in bacterial vitamin production protect both bacteria and host cells from drug toxicity, providing new opportunities for intervention and motivating the need to better understand how dietary intake and bacterial production of micronutrients like vitamin K2 influence cancer treatment outcomes.

## INTRODUCTION

With the emerging field of pharmacomicrobiomics, it is increasingly evident that bi-directional interactions exist between the gut microbiota and numerous drugs, including those not traditionally classified as antibiotics (1, 2). As such, the gut microbiota is both affected by chemotherapy and may alter chemotherapy outcomes. Treatment-related toxicity influences the quality of life of patients with colorectal cancer (CRC), often causing treatment delays and dose reductions that impact efficacy (3). Therefore, understanding the role of the gut microbiota in chemotherapy is of high clinical importance.

Capecitabine (CAP) is a commonly used chemotherapy in CRC patients, either as monotherapy or with other agents (4, 5). CAP is administered as an oral prodrug and is sequentially converted by host enzymes into active compound 5-fluorouracil (5-FU), which exerts its anticancer effects by disrupting DNA synthesis and RNA processing (6). Subsequently, 5-FU is metabolized into the inactive metabolite dihydrofluorouracil by host clearance enzyme dihydropyrimidine dehydrogenase (DPYD) (6).

CAP has two major drawbacks: limited response rates and toxicity. Despite significant advancements in early-stage CRC, overall response rates in advanced CRC remain modest, falling between 34% and 42% (7, 8). Many patients suffer from CAP-induced toxicity, with up to 57% requiring dose alterations or treatment discontinuation (9).

The gut microbiota modulates CAP efficacy and toxicity in mouse models. Specific gut bacteria harbor a bacterial homolog of host DPYD, encoded by the *preTA* operon (10, 11). *preTA*-containing bacteria can metabolize and inactivate 5-FU, modulating treatment efficacy and toxicity in mice (10, 12). Beyond direct drug metabolism, gut *Lactobacillus* potentiates CAP efficacy through immunologic and pro-apoptotic effects (13, 14).

In a clinical setting, we detected slight CAP-induced bacterial shifts in a cohort of 33 patients with advanced CRC using 16S rRNA gene sequencing (12, 15). Fecal levels of microbially-derived valerate and caproate decreased significantly during CAP treatment (16). Taken together, these studies highlighted (i) the need to measure gut microbial functional potential, (ii) the importance of mechanistic follow-up, and (iii) the utility of validation on a separate cohort. Therefore, the current study investigates CAP-microbiome interactions by performing metagenomic sequencing of stool samples from a larger cohort of advanced CRC patients with detailed toxicity data.

## RESULTS

### Prior drug use is associated with baseline microbial diversity

Fifty-six CRC patients were enrolled prior to CAP treatment (Table 1). Seventy-one percent were treated with bevacizumab in combination therapy, 70% had left-sided tumors, 29% had a colostomy, and 55% had received prior systemic treatment, mainly CAP with oxaliplatin (CAPOX; Table 1). The majority (79%) of the patients had previously undergone surgical resection of their primary tumor (Table 1).

**TABLE 1:** Baseline characteristics

Characteristic	Result
Clinical characteristics (*n* = 56)	
Age (years), mean (SD)	72.9 (7.6)
Gender, *n* (%)	
Male	36 (64.3)
Female	20 (35.7)
BMI (kg/m^2^), mean (SD)	26.77 (4.4)
Co-treatment with bevacizumab, *n* (%)	40 (71.4)
Tumor sidedness, *n* (%)	
Left-sided (descending colon, sigmoid colon, and rectum)	39 (69.6)
Right-sided (cecum, ascending colon, and transverse colon)	16 (28.6)
Missing data	1 (1.8)
Colostomy *in situ*, *n* (%)	16 (28.6)
Prior treatments and medication (*n* = 56)	
Prior systemic treatment (>1 month before inclusion),^*a*^ *n* (%)	31 (55.4)
CAP (with or without B)	6 (10.7)
CAP + RT	11 (19.6)
CAPOX (with or without B)	24 (42.9)
CAPIRI + P	1 (1.8)
FOLFIRI	2 (3.6)
FOLFIRINOX (with or without B)	2 (3.6)
TAS + B	1 (1.8)
Prior chemoradiation (>1 month before inclusion), *n* (%)	11 (19.7)
Antibiotic use last year (>3 months before inclusion), *n* (%)	23 (41.1)
Colorectal surgery in the past, *n* (%)	44 (78.6)
Proton pump inhibitor use, *n* (%)	18 (32.1)

^*a*^ CAP: capecitabine; B: bevacizumab; RT: radiotherapy; CAPOX: capecitabine + oxaliplatin: CAPIRI: capecitabine + irinotecan; P: pembrolizumab; FOLFIRI: 5-fluorouracil + irinotecan; FOLFIRINOX: 5-fluorouracil + irinotecan + oxaliplatin; TAS: trifluridine and tipiracil. Each count represents a single patient—if a patient had multiple previous rounds of a single treatment (i.e., CAPOX), this is still only counted as one event. Total percentage exceeds 100 because some patients had multiple distinct prior treatments.

**Fig 1:**
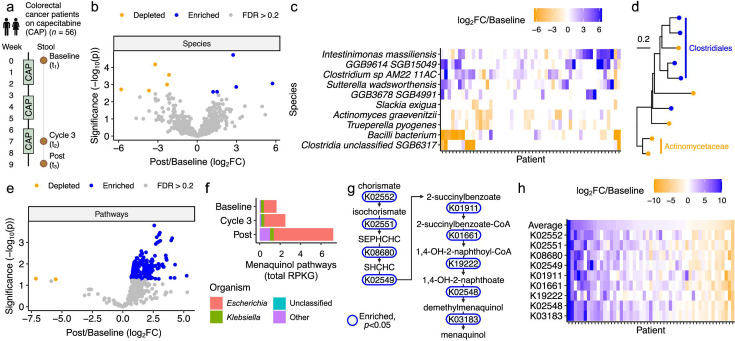
Capecitabine (CAP) alters the human gut microbiome. (**a**) Study design. Patients with advanced colorectal cancer (CRC) were treated with three cycles of CAP, with stool collected at baseline (*t*_1_), during cycle 3 (*t*_2_), and post-treatment (*t*_3_). Created with BioRender.com. (**b**) Volcano plot of species post-treatment (*t*_3_) versus baseline (*t*_1_). Points represent significantly enriched (blue) and depleted (orange) species (FDR < 0.2). (**c**) Heatmap of differentially abundant species from panel **b**, with patients and species ordered by McQuitty hierarchical clustering of log_2_ fold change (log_2_FC) of post (*t*_3_) versus baseline (*t*_1_). (**d**) Phylogenetic tree of differentially abundant species from panel **b**, with labels for clades where treatment affected multiple clade members similarly (enriched [blue] or depleted [orange]). (**e**) Volcano plot of HUMAnN 3.0 gene pathways at post (*t*_3_) versus baseline (*t*_1_). Points represent significantly enriched (blue) and depleted (orange) pathways (FDR < 0.2). Seven of the top 10 most significantly altered pathways are menaquinol biosynthesis pathways. (**f**) Genera of microbes contributing to menaquinol biosynthesis pathways. (**g**) KEGG orthologous groups (KOs) shared across all enriched menaquinol biosynthesis pathways in panel **f**. Blue indicates *P* < 0.05. (**h**) Heatmap of all KOs from panel **g**, with patients ordered by average log_2_FC (top row, “average”) and KOs ordered by occurrence in the menaquinol biosynthesis pathway. (**b, e, g**) *P*-value: mixed effects model of central log ratio (CLR)-normalized abundance versus time, with patient as a random effect.

A total of 156 stool samples were collected across three longitudinal time points (2–3 samples/subject; Fig. 1a). DNA was extracted for deep metagenomic sequencing, resulting in 39.9 ± 2.5 million high-quality sequencing reads/sample (11.7 ± 0.7 Gbp; Table S1). Inter-individual differences in microbial community accounted for 83% of the variation in the combined data set, as evidenced by species-level principal coordinate analysis (Fig. S1a).

Multiple patient characteristics were associated with variations in baseline microbial diversity and taxonomic composition. We binarized all baseline characteristics and performed *t*-tests to identify significant associations with the Shannon diversity index (Fig. S1b). Antibiotic use within the past year (>3 months before inclusion) and prior systemic treatment (>1 month before inclusion) were both associated with significantly lower baseline diversity, with most signals coming from patients who had both prior systemic treatment and prior antibiotic use (Fig. S1b through e). Next, we tested for associations between binarized patient characteristics and gut microbial community structure (Fig. S1f). Consistent with the Shannon diversity analysis, inter-individual differences in overall community structure were associated with prior antibiotic use (Fig. S1f and g).

### The human gut microbiome is altered after chemotherapy

To assess the impact of CAP treatment on the gut microbiome, we compared taxonomic and pathway abundance post-treatment (*t*_3_) relative to baseline (*t*_1_) (Fig. 1a). After adjusting for multiple hypothesis testing, we identified five enriched and five depleted species (Fig. 1b). While the overall trend was significant across the full cohort, further inspection revealed that these species were more dramatically affected in a subset of patients (Fig. 1c). The 10 differentially abundant species were from similar higher-level taxonomic groups; multiple *Clostridiales* species were enriched, while multiple *Actinomycetaceae* species were depleted (Fig. 1d; Table S2). Pathway abundance was even more dramatically altered, with 257 significantly enriched and 2 significantly depleted pathways following CAP treatment (Fig. 1e; Table S3). Taken together, these findings reveal that despite the marked heterogeneity in patient characteristics and baseline microbial community structure, it is possible to identify consistent shifts in taxonomic composition and metabolic pathway abundance following three cycles of CAP treatment.

To investigate whether these microbiome shifts would be detected during treatment, we compared taxonomic and pathway abundance during cycle 3 (*t*_2_) relative to baseline (*t*_1_). The compositional differences were more modest at this earlier time, with only two species reaching significance: *Slackia exigua* and *Clostridium* sp. NSJ 42 (Fig. S2a; Table S2). However, the overall trends were comparable, with a significant correlation in the fold change of bacterial species relative to baseline, during cycle 3, and post-treatment (Fig. S2b). Similar trends were observed in the pathway analysis. A more modest set of pathways was significantly different during treatment, including 25 enriched and 3 depleted pathways (Fig. S2c; Table S3). Nevertheless, there remained a significant correlation between pathway-level differences in relative abundance during and after treatment (Fig. S2d).

Notably, 7 of the top 10 most significantly enriched pathways post-treatment represented menaquinol biosynthesis or related pathways. Menaquinol is a reduced form of vitamin K2 (menaquinone) that is produced by diverse members of the gut microbiota and readily interconverted to menaquinone in bacterial and mammalian cells (17–19). We investigated the microbial source of menaquinol biosynthesis using stratified pathway abundance data from our patient cohort. More than 70% of menaquinol biosynthesis abundance was attributable to *Escherichia* spp., with the enrichment of *Escherichia* and unclassified sources responsible for the enrichment of menaquinol biosynthesis pathways following CAP treatment (Fig. 1f). *Escherichia* increased following CAP (Fig. S2e) and significantly explained variability in menaquinol biosynthesis abundance (Fig. S2f).

Next, we retrieved the KEGG orthologous groups (KOs) shared across all seven enriched menaquinol biosynthesis pathways. All of these KOs were significantly enriched (Fig. 1g). Analysis at a per-patient level revealed clear inter-individual differences in the temporal shifts in menaquinol biosynthesis pathway relative abundance, with 70.5% of patients exhibiting a net increase relative to baseline (Fig. 1h). Patients who experienced menaquinol biosynthesis gene enrichment had significantly lower-stage disease at diagnosis (Fig. S2g) and were significantly more likely to require dose reductions during treatment (Fig. S2h).

### Menaquinol biosynthesis rescues bacterial fluoropyrimidine sensitivity

Because bacterial menaquinol biosynthesis genes were enriched following fluoropyrimidine treatment and are responsible for the production of menaquinones (vitamin K2) (18), we hypothesized that menaquinol biosynthesis could be a protective factor allowing bacteria to escape the off-target effects of fluoropyrimidines on gut bacteria (10). The model organism *Escherichia coli* K-12 encodes an intact menaquinol biosynthesis pathway (20), is sensitive to fluoropyrimidines (10), and is genetically tractable (21), providing a useful model system to test causal links between vitamin K2 production, anticancer drugs, and bacterial growth.

We leveraged a previously published genome-wide random barcode transposon-site sequencing (RB-TnSeq) library that covers 3,728 non-essential genes with a total of 152,018 unique transposon insertions (22). The *E. coli* RB-TnSeq library was cultured for 48 hours in M9 minimal media with vehicle or 500 µM of three fluoropyrimidines that had all been previously shown to inhibit *E. coli* growth (10): CAP, 5′-deoxy-5-fluorocytidine (DFCR), and 5-FU.

**Fig 2:**
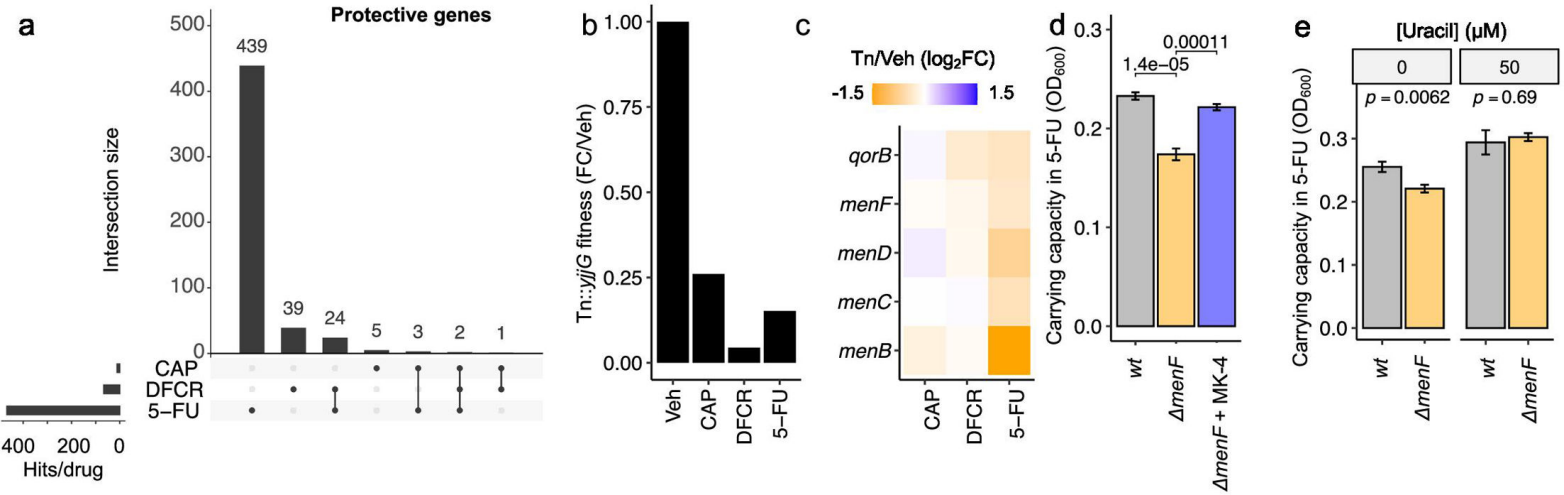
Menaquinol biosynthesis rescues bacterial sensitivity to fluoropyrimidines. (**a–c**) An *E. coli* RB-TnSeq library was treated with 500 µM of capecitabine (CAP), 5′-deoxy-5-fluorocytidine (DFCR), 5-fluorouracil (5-FU), or vehicle (Veh) in duplicate for 48 hours. (**a**) Upset plot of significantly depleted transposon-disrupted genes (intact gene is protective) across all three conditions. (**b**) Fitness of Tn::*yjjG* mutant in all four conditions, relative to vehicle. Values represent the mean of two biological replicates. (**c**) Gene set enrichment analysis of protective genes from panel **a** revealed quinone biosynthesis as the sole significantly enriched pathway (hypergeometric *P* < 0.01). RB-TnSeq fold changes of enriched protective quinone biosynthesis genes are depicted. (**d and e**) *E. coli* BW25113 wild-type (*wt*) and Δ*menF::Kan^R^* (Δ*menF*) were treated with 500 µM 5-FU ± 225 nM menaquinone (MK-4) (**d**) or ± 50 µM uracil (**e**) for 24 hours, with carrying capacity quantified with Growthcurver. *P*-values: deviation from linearity on quantile-quantile plot (**a**), Student’s *t*-test (**d and e**).

5-FU induced major overall changes in library composition (Fig. 2a; Fig. S3a and b; Tables S4 to S6). We identified a total of 513 protective (Fig. 2a) and 274 detrimental (Fig. S3b) genes during incubation with any of the three fluoropyrimidines. A subset of genes was consistent across the three drugs, including two protective and two detrimental genes (Tables S4 to S6). Transposon insertions in the uracil phosphoribosyltransferase (*upp*) gene were dramatically enriched in response to 5-FU and DFCR (Fig. S3c), confirming its key role in exacerbating bacterial 5-FU toxicity (23). On the other hand, dUMP phosphatase (*yjjG*) insertions were dramatically depleted across all conditions (Fig. 2b), confirming its role in mitigating bacterial 5-FU toxicity by preventing incorporation of mutagenic nucleotides (24).

Next, we performed gene set enrichment analysis for genes that were enriched or depleted by at least one drug to gain a high-level view of the genetic determinants of fluoropyrimidine sensitivity. The detrimental genes in response to fluoropyrimidine treatment (5-FU, DFCR, and/or CAP) were significantly enriched for homologous recombination (*P* = 0.0099; Fig. S3d), including transposon insertions in Holliday junction DNA helicase *ruvA/ruvB*, potentially due to enhanced cellular toxicity following inaccurate DNA damage repair. Protective genes in response to fluoropyrimidines were significantly enriched only for quinone biosynthesis (*P* = 0.0056), including many of our previously identified genes for menaquinol biosynthesis (Fig. 1g). Consistent with the broader pattern in this analysis, 5-FU led to a more marked depletion of menaquinol biosynthesis transposon insertions (Fig. 2c).

Bacterial genetics and media supplementation validated a causal role of menaquinol biosynthesis in mediating protection from the off-target effects of fluoropyrimidines for bacterial growth. First, we acquired an in-frame, kanamycin (Kan) resistant single gene deletion of the first step of the menaquinol biosynthesis pathway (*menF*, K02552) from the Keio collection (25). We grew *E. coli* BW25113 wild-type (*wt*) and Δ*menF::Kan^R^* in 0 and 500 µM 5-FU. Overall growth of the two strains was comparable in the absence of 5-FU (Table S7). The carrying capacity of Δ*menF::Kan^R^* relative to *wt* decreased when subjected to 5-FU (Fig. 2d). Next, we grew *E. coli* BW25113 Δ*menF::Kan^R^* in 5-FU with 225 nM menaquinone. Menaquinone markedly rescued carrying capacity in the presence of 5-FU (Fig. 2d; Table S7).

Prior studies showed that menaquinol biosynthesis defects lead to uracil auxotrophy in *E. coli* (26–28), suggesting this pathway may exert a chemoprotective effect via modulating uracil. To test whether uracil could rescue the 5-FU-dependent Δ*menF::Kan^R^* fitness defect, we grew *E. coli wt* and Δ*menF::Kan^R^* in 5-FU ± 50 µM uracil. While Δ*menF::Kan^R^* grew worse than *wt* in 5-FU in media, both strains grew comparably with uracil supplementation (Fig. 2e; Table S7). Taken together, these findings suggest that fluoropyrimidines directly select for bacteria with the ability to synthesize chemoprotective menaquinone, prompting us to consider the broader chemoprotective role of the microbiome in mediating host drug toxicities.

### Baseline gut microbial functional pathways are associated with drug toxicities

**Fig 3:**
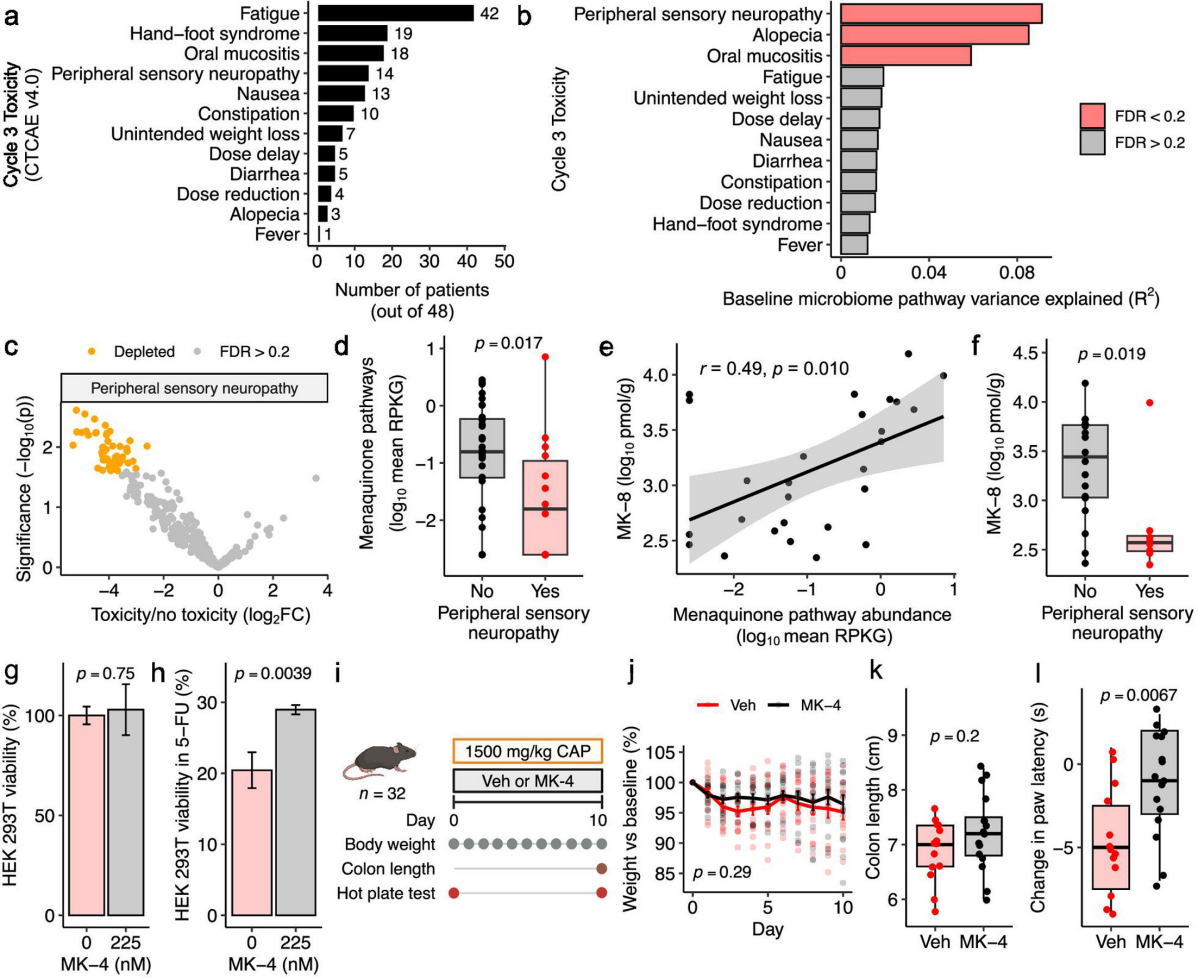
Pre-treatment microbial gene pathways are associated with the development of toxicities during treatment. (**a**) Distribution of Grade 1+ toxicities in patients at cycle 3 (*t*_2_). (**b**) Permutational multivariate analysis of variance (PERMANOVA) testing of cycle 3 (*t*_2_) toxicities with respect to baseline bacterial gene family composition. *P-*value: PERMANOVA test using the central log ratio (CLR)-transformed Euclidean metric of baseline bacterial pathway composition, with FDR calculated with Benjamini-Hochberg multiple-testing correction. (**c**) Volcano plot of baseline gene pathways in patients who went on to have peripheral sensory neuropathy (PSN) or no PSN during treatment. Colored points represent significantly depleted (orange) pathways (FDR < 0.2). *P*-value: linear model of abundance versus toxicity. Five of the top 10 most significantly altered pathways are menaquinol biosynthesis superpathways. (**d**) Baseline menaquinone pathway gene abundance versus *t*_2_ PSN. *P*-value: Wilcoxon rank-sum test. (**e**) Menaquinone pathway gene abundance versus stool menaquinone-8 (MK-8) metabolite abundance. *R*, *P*-value: Pearson’s correlation. (**f**) Baseline stool MK-8 metabolite abundance versus *t*_2_ PSN. *P*-value: Wilcoxon rank-sum test. (**g and h**) HEK 293T cells were incubated for 48 hours ±225 nM menaquinone (MK-4) in the absence (**g**) and presence (**h**) of 75 µM 5-FU, with viability measured by MTT assay and normalized to cells grown in MK-4-free, 5-FU-free media. (**i–l**) Thirty-two mixed-sex mice were treated with 1,500 mg/kg capecitabine (CAP) ± 40 mg/kg MK-4 by oral gavage daily for 10 days, with body weight measured daily, colon length measured on day 10, and paw latency at 52°C hot plate test measured on days 0 and 10. (**i**) Experimental design schematic. (**j**) Body weights. (**k**) Endpoint colon length. (**l**) Per-mouse change in paw withdrawal latency between day 0 and day 10. Negative values correspond with more severe paw sensitivity. *P*-values: Mann-Whitney *U*-test (**g, h, k**), two-way ANOVA (**j**), Wilcoxon signed-rank test (**l**). Sample size: patients with at least one documented *t*_2_ toxicity event (*n* = 48; **a and b**); patients with documented PSN status (*n* = 47; **c and d**); patients with stool metabolite data (*n* = 27; **e**); patients with stool metabolite data and documented PSN status (*n* = 26; **f**); biological replicate wells (*n* = 6/group; **g and h**); mice (*n* = 32; **i–l**).

Most patients experienced at least one patient-reported toxicity-related event (any grade) during treatment (*n* = 45/48 patients with *t*_2_ toxicity data available; Fig. 3a). To investigate whether the microbiome varies by toxicity status, we performed PERMANOVA testing comparing these on-treatment toxicities with the community composition of baseline species and pathway abundances. We did not find any significant relationships with baseline species abundance (FDR > 0.2). In contrast, the composition of baseline pathway abundance was significantly associated with neuropathic pain (peripheral sensory neuropathy [PSN]), alopecia (hair loss), and oral mucositis (Fig. 3b).

Next, we compared baseline pathway abundance in patients who went on to report PSN-like symptoms, alopecia, and oral mucositis, and those who did not. We found a significant depletion in 59 pathways in patients who went on to experience PSN, with 3 of the top 10 most significantly affected pathways involving menaquinol biosynthesis (Fig. 3c; Table S8). Remarkably, patients who reported PSN-like symptoms had significantly lower baseline levels of menaquinol biosynthesis pathways (Fig. 3d), supporting the potential clinical relevance of our microbiome analyses (Fig. 1) and experiments in bacterial cultures (Fig. 2). We further validated these findings using mass spectrometry to measure phylloquinone and menaquinones in stool samples (Fig. S4a). Baseline levels of stool menaquinone-8 (MK-8) were significantly associated with menaquinone biosynthesis pathway abundance (Fig. 3e; Fig. S4b). Baseline MK-8 levels were significantly lower in patients who went on to experience PSN-like symptoms (Fig. 3f). Further analysis of the cycle 3 and post-treatment pathway data revealed that baseline differences in PSN-associated pathways equalize during treatment (Fig. S5a).

Distinct pathways were observed for alopecia, with a significant depletion of 291 pathways at baseline in patients who experienced alopecia (Fig. S5b; Table S9). We did not observe a role for menaquinol biosynthesis pathways (Table S9). Instead, we noted a depletion in pathways involved in l-methionine biosynthesis and β-(14)-mannan degradation (Fig. S5c). Oral mucositis was not associated with any individual pathways (FDR > 0.2). None of the measured patient characteristics (Table 1) were significantly associated with PSN, alopecia, or oral mucositis (Fisher’s exact *P* > 0.05).

*In vitro* and *in vivo* toxicity studies suggest a causal role of menaquinone in protecting against PSN. Since HEK 293T sensitivity reflects pharmacologic neurotoxicity (29), we treated HEK 293T cells with varying levels of 5-FU and MK-4 for 48 hours and assessed endpoint viability (Table S10). While MK-4 did not significantly impact viability in the absence of 5-FU (Fig. 3g), MK-4 partially rescued cell viability in the presence of 5-FU (Fig. 3h). To assess the *in vivo* significance of these findings, we treated 32 mixed-sex C57BL/6J mice with 1,500 mg/kg CAP with or without 40 mg/kg MK-4 (Fig. 3i). MK-4 did not significantly impact mouse weight loss (a marker of systemic toxicity; Fig. 3j) or colon length (a marker of gastrointestinal inflammation; Fig. 3k). However, MK-4 supplementation was sufficient to rescue CAP-induced thermal hind paw hyperalgesia (Fig. 3l), an indicator of PSN in mice (30).

Finally, we analyzed the bacterial *preTA* operon, which encodes an enzyme that inactivates 5-FU to its downstream metabolite dihydrofluorouracil (10). Consistent with the U.S. cohort (12), *preTA* was significantly increased following CAP treatment in patients in the Netherlands cohort (Fig. S6a). *preA* (b2145) was identified as a protective gene in our RB-TnSeq analysis (Table S4). Baseline levels of *preTA* were positively associated with fewer required dose adjustments (Fig. S6b), but not significantly associated with PSN (Fig. S6c), hand-foot syndrome (HFS) (Fig. S6d), or drug efficacy (Fig. S6e and f).

### Baseline gut microbial gene family abundance accurately predicts toxicity

**Fig 4:**
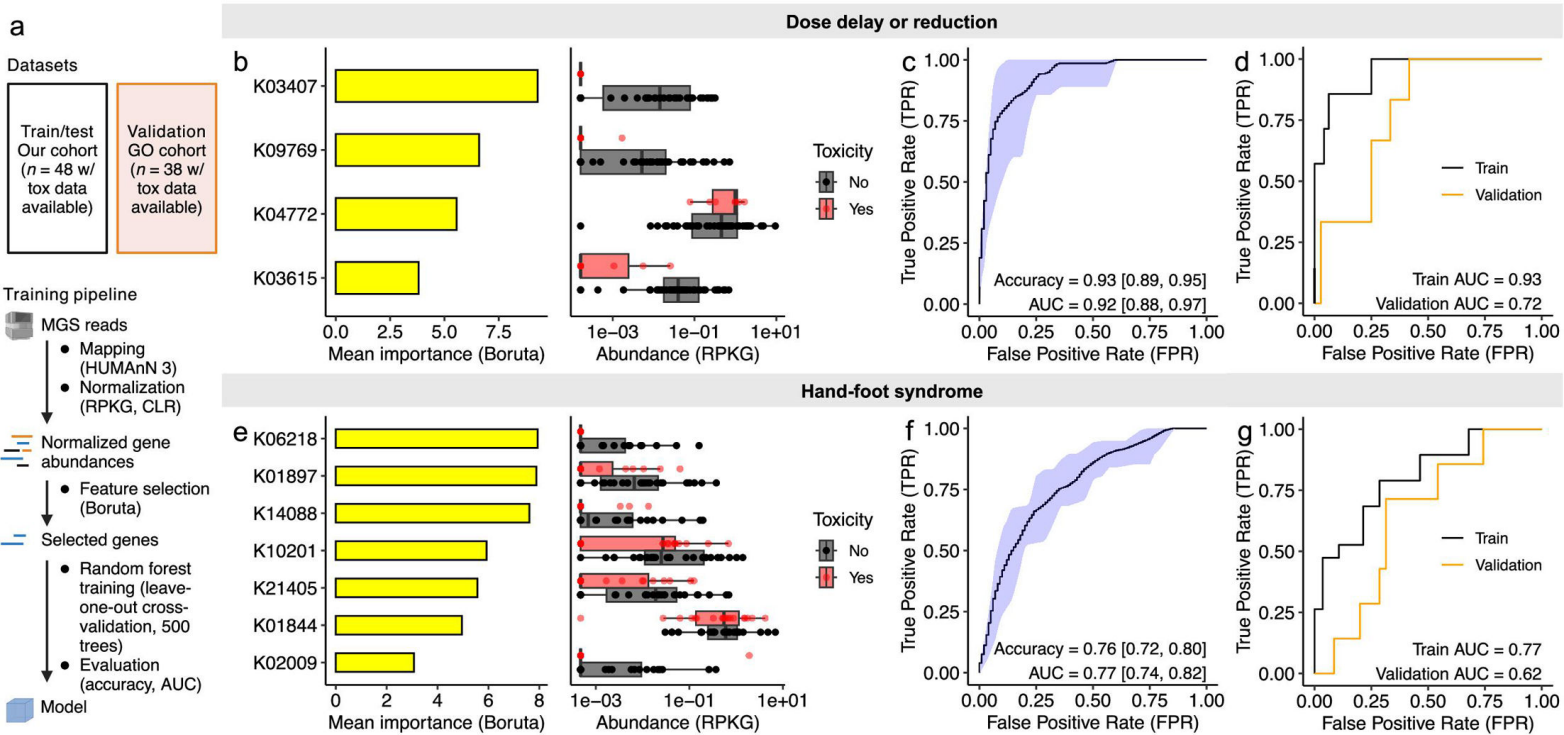
The baseline gut microbiome predicts drug side effect profiles. (**a**) Random forest pipeline. For each toxicity of interest, metagenomic sequencing reads were mapped to KEGG orthologous groups (KOs) using HUMAnN 3 and normalized as reads per kilobase per genome equivalent (RPKG), followed by a central log ratio (CLR) transform, followed by feature selection with Boruta. A random forest algorithm was trained on these features using leave-one-out cross-validation (LOOCV) with 500 trees, followed by evaluation on our cohort and an independent validation cohort of 38 American patients with toxicity data available (12). Created with BioRender.com. (**b and e**) Importance scores and baseline (*t*_1_) abundances of Boruta-selected KOs to classify dosing changes (**b**) or hand-foot syndrome (HFS) (**e**) during treatment (*t*_2_). (**c and f**) Receiver operating characteristic (ROC) curve for classification of dosing changes (**c**) or HFS (**f**) with random forest models built with Boruta-selected KOs tested with LOOCV. The black line represents the mean, and the blue shaded area represents the 95% confidence interval obtained across 100 independent models. Accuracy and area under the curve (AUC) are displayed, with 95% confidence intervals in brackets. (**d and g**) Evaluation of a model trained on our data set and validated on an independent cohort of 38 American patients to predict dosing changes (**d**) or HFS (**g**).

We sought to build a model using the baseline microbiome to predict toxicity during CAP treatment. Rather than relying on gene pathways that encompass genes with broad functions, we turned to more granular KO abundance data. For each toxicity of interest, we used Boruta to select KOs of interest, trained a random forest model on this cohort (47–55 patients with on-treatment toxicity data available), and validated the model on an independent cohort (38 patients with on-treatment toxicity data available; Fig. 4a). The validation cohort consisted of fluoropyrimidine-treated patients with CRC treated at the University of California, San Francisco (ClinicalStudies.gov NCT04054908) (12). Due to the availability of detailed HFS (any grade) and dose adjustment data in the validation clinical data set, we opted to focus on these toxicity categories.

In our training cohort, the Boruta algorithm selected four baseline microbial KOs as features relevant to the development of a model predicting dose adjustments (Fig. 4b). Of these KOs, serine endoprotease *degQ* (K04772) was more abundant in dose-adjusted patients, while sensor kinase *cheA* (K03407), nucleotide metabolism esterase *ymdB* (K09769), and ion-translating oxidoreductase *rnfC* (K03615) were more abundant in patients who did not require dose delays or reductions (Fig. 4b). Using these four genes, we trained and tested 100 random forest models using leave-one-out cross-validation in our training cohort, achieving a mean accuracy of 0.93 and the area under the curve (AUC) of 0.92 (Fig. 4c). Finally, we applied a random forest model trained on our full training cohort to our validation cohort, obtaining an AUC of 0.72 (Fig. 4d).

For HFS, the Boruta algorithm selected seven baseline microbial KOs as relevant features in our training cohort (Fig. 4e). All of these KOs were less abundant in patients who experienced HFS (Fig. 4e). Using these seven genes, we trained 100 random forest models using leave-one-out cross-validation, achieving a mean accuracy of 0.76 and AUC of 0.77 (Fig. 4f). The validation cohort AUC was 0.62 (Fig. 4g).

Finally, inspired by our observations associating baseline pathway abundance and PSN, we sought to develop an algorithm to predict patient-reported PSN in our cohort in spite of a lack of validation cohort for this toxicity. We selected the top 10 differentially abundant pathways (Fig. 3c and d) and trained 100 random forest models using leave-one-out cross-validation, achieving a mean accuracy of 0.77 and AUC of 0.72 (Fig. S7a). We then trained three simpler generalized linear models using leave-one-out cross-validation: Model 1 (baseline menaquinone abundance), Model 2 (delta menaquinone abundance, baseline vs on-treatment), and Model 3 (both baseline and delta menaquinone abundance). Of these, Model 3 achieved the highest AUC (0.71; Fig. S7b). Consistent with this modeling, for patients with low baseline menaquinone, menaquinone expansion during treatment was associated with protection from PSN (Fig. S7c) but not associated with different rates of dose adjustments (Fig. S7d). Adding menaquinol biosynthesis genes to Boruta-selected features did not markedly improve prediction of dose adjustments or HFS (Table S11), and menaquinol biosynthesis genes alone poorly predicted dose adjustments and HFS (Table S11). Menaquinol biosynthesis pathway abundance was not associated with tumor response in either patient cohort (Fig. S8).

## DISCUSSION

Our metagenomic and experimental data revealed an unexpected role for microbial vitamin K2 biosynthesis in protection from the off-target effects of fluoropyrimidines on gut bacterial growth. Bacteria are exposed to 5-FU in the intestinal lumen (6, 12, 31), likely due to a combination of microbial bioactivation of CAP (32) and nucleotide transporter-mediated 5-FU export from intestinal and tumor cells (33). The primary mechanism of action of 5-FU, thymidylate synthase inhibition, does not have any direct links to vitamin K2, in contrast to other micronutrients including folate and vitamin B6 (34, 35). Our experiments in *E. coli* suggest this pathway may exert a chemoprotective effect via modulating uracil, protecting bacteria from 5-FU (10).

These data support the protective role of microbial menaquinol biosynthesis in ameliorating aspects of host drug toxicity. Higher baseline menaquinol biosynthesis gene and menaquinone-8 levels were associated with decreased risk of patient-reported PSN, and menaquinone-4 (MK-4) protected against neurotoxicity in mammalian cell lines and mice. Consistent with this data, demyelination of peripheral nerve fibers is a primary cause of PSN (36); menaquinones including MK-4 and MK-7 play a crucial role in the myelin sheath repair in the peripheral nervous system (37, 38). Supplementation with MK-7 can alleviate peripheral neuropathies in patients with vitamin B12 deficiency or type 2 diabetes mellitus (39). While human supplementation studies often use MK-7 for its high bioavailability (40), low MK-7 and MK-8 levels are associated with similar pathophysiologies (41–43), highlighting the need for direct comparisons of MK-7 and MK-8 supplementation in preclinical and clinical studies.

More broadly, we identified microbial biomarkers of drug toxicity across multiple endpoints (PSN, alopecia, and oral mucositis). We found lower levels of mannan degradation genes in the gut microbiomes of subjects who developed alopecia. Intraperitoneal mannan delivery induced alopecia in a mouse model (44). Thus, the balance between mannan consumption through diet, fungal production within the gastrointestinal tract, and gut bacterial degradation may modulate alopecia through systemic mannan levels.

Our data provides a proof-of-concept for the development of microbiome-based machine learning models that accurately predict drug toxicity in cancer chemotherapy patients, building upon prior studies in rheumatoid arthritis, prostate cancer radiotherapy, and immune checkpoint inhibitor-induced colitis (45–48). Remarkably, these models required just four to seven KOs, which could be measured using less expensive and more rapid targeted assays like quantitative PCR. A critical next step will be designing larger intervention studies to test the utility of such models in clinical decision-making.

The current data set has multiple limitations to address in subsequent efforts. We did not collect samples during the first two treatment cycles, potentially missing dramatic early-treatment shifts observed in a cohort of U.S. CRC patients (12). While our sample size (56 subjects, 156 samples) was sufficient to reach statistical significance and uncover interesting biology, it remains insufficient to inform concrete patient care guidelines. This is especially relevant for our alopecia findings: only three patients in our study developed on-treatment alopecia, consistent with alopecia rates ~5% in CAP-treated patients in prior studies (49). The observational nature of our study and lack of dietary data make causal inferences challenging, a limitation partially overcome by our experimental validation.

Many patients in this cohort reported PSN-like symptoms, a toxicity more commonly associated with oxaliplatin than with CAP. The reported PSN-like symptoms may be related to HFS, a dose-limiting toxicity of CAP, which initially manifests with discomfort and tingling similar to neuropathy. Alternatively, CAP might worsen subclinical PSN in patients previously treated with oxaliplatin (50). In our cohort, only 8 out of 14 patients reporting PSN had previous oxaliplatin exposure. Although PSN is less common than HFS, previous studies have also observed PSN in 16–37% of patients treated with CAP or 5-FU without oxaliplatin (51, 52).

In conclusion, our findings provide further support for the role of the gut microbiome in mediating cancer treatment outcomes and the utility of paired studies in well-characterized patient cohorts and experimental model systems. These results raise numerous testable hypotheses that should be explored in preclinical models. Future work should focus on controlled clinical intervention studies to investigate if the use of vitamin supplementation, probiotics, or other microbiome-based interventions can alleviate drug toxicity in cancer patients.

## MATERIALS AND METHODS

Resources utilized in this study are listed in Table 2.

**TABLE 2:** Resources used for this study

Reagent or resource	Source	Identifier
Experimental models: bacterial strains, cell lines, mice		
*E. coli* RB-TnSeq library	Reference 53	Provided by Deutschbauer Lab
*E. coli* BW25113 wild type	Reference 25	KEIO Collection
*E. coli* BW25113 Δ*menF::KanR*	Reference 25	KEIO Collection
HEK 293T cell line	Reference 54	Provided by Mukherjee Lab
C57BL/6J mice	The Jackson Laboratory	000664
Chemicals, peptides, and recombinant proteins		
Luria broth	Millipore Sigma	L3152
Brain heart infusion	Fisher Scientific	237500
Kanamycin	Millipore Sigma	K1377
5-Fluorouracil	Millipore Sigma	343922
Capecitabine	Fisher Scientific	50148375
5′-Deoxy-5-fluorocytidine	Santa Cruz Biotechnology	221055
Uracil	Millipore Sigma	U0750
MK-4	Millipore Sigma	V9378
Phylloquinone	Millipore Sigma	95271
MK-5 to MK-13 standards	Hoffman-LaRoche and Co. (former)	Provided by Fu Lab
HPLC-grade solvents	Fisher Scientific	Various
High-glucose DMEM	UCSF Media Production Facility	CCFAA005
GlutaMAX	Gibco	35050061
Heat inactivated fetal bovine serum	Fisher Scientific	A5256801
Penicillin-streptomycin	Life Technologies	15140122
Clinical surveys		
CTCAE v4.0	National Institutes of Health	https://ctep.cancer.gov/protocoldevelopment/electronic_applications/ctc.htm#ctc_40
Deposited data		
Raw sequencing data with human reads removed	This study	PRJNA1171107
Commercial kits		
ZymoBIOMICs 96 MagBead DNA Kit	Zymo Research	D4302
Illumina DNA Prep (M) Tagmentation (96 Samples, IPB)	Illumina	20060059
Quant-iT Picogreen dsDNA Kit	Invitrogen	P7589
MTT Cell Proliferation Assay Kit	Cayman Chemical	10009365
Instruments		
Mini-Beadbeater-96	BioSpec	1001
Anaerobic chamber	Coy Laboratory Products	1200001
High Performance Microplate Spectrophotometer	BioTek	EON
NovaSeq 6000	Illumina	3376672
Agilent 6130 Quadrupole LC/MS	Agilent	9909
Software and algorithms		
All code for plots and statistical analysis	This study	https://github.com/turnbaughlab/2024_Trepka_DrugToxicity
MetaPhlAn 4	Reference 55	https://github.com/biobakery/MetaPhlAn
HUMAnN 3	Reference 56	https://github.com/biobakery/humann
R v4.2.1	Reference 57	https://r-project.org/
microbeCensus 1.0.4	Reference 58	https://github.com/snayfach/MicrobeCensus
vegan 2.6.8	Reference 59	https://cran.r-project.org/web/packages/vegan/index.html
ggtree 3.15.0	Reference 60	https://github.com/YuLab-SMU/ggtree
ggplot2 3.5.1	Reference 61	https://cran.r-project.org/web/packages/ggplot2/index.html
ggpubr 0.6.0	Reference 62	https://cran.r-project.org/web/packages/ggpubr/index.html
Boruta 8.0.0	Reference 63	https://cran.r-project.org/web/packages/Boruta/index.html
randomForest 4.7.1.2	Reference 64	https://cran.r-project.org/web/packages/randomForest/index.html
caret 6.0.94	Reference 65	https://cran.r-project.org/web/packages/caret/index.html
pROC 1.18.5	Reference 66	https://cran.r-project.org/web/packages/pROC/index.html
Other		
Preservation-free tubes for stool collection	Sarstedt	80.623.022
BreatheEasy Covers	Millipore Sigma	Z380059

### Study design and population

This prospective longitudinal cohort study was conducted at Maastricht University Medical Center (MUMC+), Catharina Hospital Eindhoven, Hospital Gelderse Vallei, and VieCuri Medical Center in the Netherlands, in accordance with the previously published study protocol (NL-OMON29314/NTR6957) (67). Patients were eligible if diagnosed with metastatic or unresectable CRC with planned CAP treatment, with or without bevacizumab. Exclusion criteria included radiotherapy within 2 weeks of enrollment, other systemic therapy within 1 month of enrollment, antibiotic use within 3 months of enrollment, microsatellite instability, creatinine clearance <30 mL/min, and (sub)total colectomy and/or ileostomy. CAP was administered in a 3-week cycle of 2 weeks of oral CAP at a dose of 1,000–1,250 mg/m² twice daily, followed by 1 week of rest. Treatment was adjusted if deemed necessary by the treating oncologist.

### Sample and data collection

#### Fecal samples

Fecal samples were collected by patients at home in preservation-free tubes and stored in freezers at three time points: before CAP initiation (*t*_1_), during week 2 of CAP cycle 3 (*t*_2_), and after week 3 of cycle 3 (*t*_3_). Frozen samples were transported to the hospital in cooled containers and stored at −80°C long term.

#### Clinical data and chemotherapy-induced toxicity

Patients completed questionnaires on health-related characteristics and medical history. Chemotherapy-induced toxicities were self-reported by patients and scored based on Common Terminology Criteria for Adverse Events (CTCAE v4.0) (https://ctep.cancer.gov/protocoldevelopment/electronic_applications/ctc.htm). The questionnaire encompassed nausea (0–3), vomiting (0–5), diarrhea (0–5), unintended weight loss (0–3) constipation (0–5), PSN (0–5), oral mucositis (0–5), HFS (0–3), fever (0–5), alopecia (0–2), and fatigue (0–3). Additional data about medical history, tumor characteristics, medications, surgery, DPYD deficiency, and CAP dose adjustments were collected from medical records.

### Gut microbiome analysis

ZymoBIOMICs 96 MagBead DNA Kit was used for fecal DNA extractions (156 samples from 56 patients), with extraction, library preparation, quality control, sequencing, and read mapping performed with Illumina Tagmentation and Picogreen kits as described previously (12). Taxa abundances were central log ratio (CLR)-transformed. Pathway/gene abundances were normalized to reads per kilobase per genome equivalent using microbeCensus (58). Shannon diversity was calculated using the vegan command diversity (59). PERMANOVA was performed using vegan commands vegdist (CLR-Euclidean/Aitchison distance) and adonis2 (59) to compare patient demographics and patient-reported toxicities (any grade) to the baseline microbiome. Differential abundance was calculated using linear mixed-effects modeling with time as a fixed effect and patient as a random effect (nlme command lme), followed by Benjamini-Hochberg correction. Phylogenetic trees were obtained by pruning the MetaPhlAn 4 tree (55) and visualized using ggtree (60).

### Vitamin K analysis

*t*_1_ stool samples with >75 mg wet stool remaining were dried by lyophilization. Menaquinones and phylloquinone in freeze-dried stool were extracted and quantified by LC-MS as previously described with the following limits of detection (LOD): 1 pmol/g MK-10; 5 pmol/g MK-5, MK-7, MK-8, MK-9, MK-11, MK-12, and MK-13, 10 pmol/g MK-6; 30 pmol/g PK and MK-4 (68).

### *In vitro* studies of fluoropyrimidine toxicity in *E. coli*

#### Transposon sequencing experiment

We performed *E. coli* transposon mutant fitness assays as described previously (53). A thawed transposon library aliquot was grown overnight in 25 mL Luria broth (LB) with 50 µg/mL kanamycin at 37°C with 225 rpm shaking. Cells were then inoculated into competitive growth assays in fluoropyrimidines (500 µM CAP, 5-FU, and DFCR) or vehicle. Assays were performed in duplicate in 200 µL M9 minimal media with starting OD_600_ = 0.02. After 48 h, cell pellets were collected and gDNA extracted with ZymoBIOMICS 96 MagBead DNA Kit, according to the manufacturer’s protocol. We performed barcode sequencing as previously described, averaging independent insertions at the gene level and calculating log-ratios (22). A quantile-quantile method was used to determine significance [abs(ln(FC)) > 0.25, abs(log(VehFitness)) < 0.05; Fig. S3a]. Overlap between conditions was visualized using UpSetR (69). Gene set enrichment analysis was performed using the clusterProfiler function enrichKEGG (universe = library, organism = “eco,” pvalueCutoff = 0.01) (70).

#### 5-FU sensitivity experiments

*E. coli* BW25113 wild-type and Δ*menF::Kan^R^* were obtained from the Keio collection (25) and streaked on LB with 30 µg/mL kanamycin. Colonies were subcultured overnight in brain heart infusion (BHI) in an anaerobic chamber at 37°C with an atmosphere of 3% H_2_, 20% CO_2_, and balance N_2_. 5-FU was assayed at 0 and 500 µM. While *E. coli* primarily produces long-chain menaquinone MK-8, we used shorter-chain MK-4 for supplementation given its wide-reaching ability to functionally rescue enzymatic and cellular processes in prior work (27, 28, 71–73). MK-4 was dissolved in methanol, supplemented 1% (vol/vol; 2 µL MK-4 solution/200 µL cell media), and assayed at 0 and 225 nM (0 and 0.1 µg/mL). Uracil was assayed at 0 and 50 µM. A total of 3 µL seed culture diluted to OD_600_ = 0.1 was inoculated with 197 µL media ± drug in a 96-well plate. Plates were covered and incubated anaerobically at 37°C for 24 h in a plate reader, with 1 min linear shake prior to OD_600_ readings every 15 min. Carrying capacity was determined using the package Growthcurver (74).

### *In vitro* studies of 5-FU toxicity in mammalian cells

HEK 293T cells were passaged in high-glucose DMEM supplemented with 10% FBS, 2 mM GlutaMAX, and 50 U penicillin-streptomycin, with cells split at 70–80% confluence and used between passages 3 and 8 as described previously (54). For toxicity studies, cells were seeded in non-edge wells of a 96-well plate at 10^4^ cells/well. 5-FU and MK-4 stock solutions (100×) were prepared in water (5-FU) or ethanol (MK-4) and filter sterilized. Cells were incubated for 48 h with 98 µL media, 1 µL 5-FU solution, and 1 µL MK-4 solution for final concentrations of 0, 225 nM, 2.25 µM MK-4 and 0, 7.5, and 75 µM 5-FU, with six biological replicates per 5-FU/MK-4 concentration pair. Cell viability was measured using the MTT assay according to the manufacturer’s protocol, with background subtraction of cell-free wells and percent viability calculated relative to 5-FU-free, MK-4-free wells.

### *In vivo* studies of CAP toxicity

Thirty-two mixed-sex C57BL/6J mice (16 per independent experiment) were housed 4/cage and gavaged daily for 10 days with 1,500 mg/kg CAP in citrate buffer with 5% gum arabic as described previously (12). For *n* = 16 mice, 40 mg/kg MK-4 was dissolved in the CAP solution for delivery. Mouse weight was measured daily. Colon length was measured at the endpoint. A 52°C hot plate test measuring time to hind paw lick, flick, or jump was performed at baseline and endpoint as a proxy for peripheral neuropathy (30).

### Random forest modeling

Two cohorts were used for modeling: a training cohort (this Dutch cohort, 47–55 patients with baseline stool sequencing and cycle 3 toxicity data for HFS [*n* = 47] or dose adjustments [*n* = 55]), and an independent validation cohort (U.S. cohort, *n* = 38 patients with baseline stool sequencing and on-treatment toxicity data) (12). HFS and dose adjustment were selected as targets due to available toxicity data in both cohorts, while PSN was chosen due to strong microbiome signals in our analysis. Features (CLR-normalized KOs) were selected by applying the Boruta algorithm (63) to the training cohort for HFS and dose adjustments. For PSN, the top 10 pathways from differential abundance testing were used as features, with no external validation cohort (no PSN data for U.S. cohort). A random forest model was fitted using these features (500 trees, leave-one-out cross-validation [LOOCV]) using packages randomForest (64) and caret (65). Within-cohort model accuracy was evaluated by training 100 separate models, validating on left-out samples (LOOCV), and plotting the mean and 95% confidence interval of receiver operating curves using pROC (66). For HFS and dose adjustments, model generalizability was validated by applying a model trained on the full Dutch cohort to the U.S. validation cohort.

### Statistical analysis

Statistical analysis was performed in R (v4.2.1) (57), with plots generated using ggplot2 (v3.5.1) and ggpubr (v0.6.0) (61, 62). Statistical tests are specified in the text/figure legends and summarized here. PERMANOVA (CLR-Euclidean ordination) was used to test compositional differences in taxa vs patient characteristics and gene pathways vs toxicity. Linear mixed-effects modeling (time as fixed effect, patient as random effect) was used to identify time-dependent changes in taxa/genes. *t*-tests, Spearman’s correlation, and likelihood-ratio tests were used to identify relationships between categorical/continuous, continuous/continuous, and categorical/categorical variables, respectively. Significance was determined as *P* < 0.05 (individual tests) or Benjamini-Hochberg FDR < 0.2 (multiple hypothesis correction).

## Data Availability

This study did not generate new unique reagents. Raw sequencing data with human reads removed have been deposited to the NCBI Sequence Read Archive (BioProject PRJNA1171107). Processed data sets and all original code used in this study are available on GitHub (https://github.com/turnbaughlab/2025_Trepka_DrugToxicity).

## SUPPLEMENTAL MATERIAL

Supplemental Figures mbio.00930-25-s0001.pdf Figures S1 to S8.

Supplemental Tables mbio.00930-25-s0002.xlsx Tables S1 to S11.

ASM does not own the copyrights to Supplemental Material that may be linked to, or accessed through, an article. The authors have granted ASM a non-exclusive, world-wide license to publish the Supplemental Material files. Please contact the corresponding author directly for reuse.
