# Supplementary material for: Microbial vitamin biosynthesis links gut microbiota dynamics to chemotherapy toxicity
Source: mBio. 2025 May 20;16(6):e00930-25. doi: 10.1128/mbio.00930-25 (PMC12153289; doi:10.1128/mbio.00930-25)
Supplement: Supplemental Figures — Figures S1 to S8. [file mbio.00930-25-s0001.pdf]

Supplemental Figures and Figure Legends

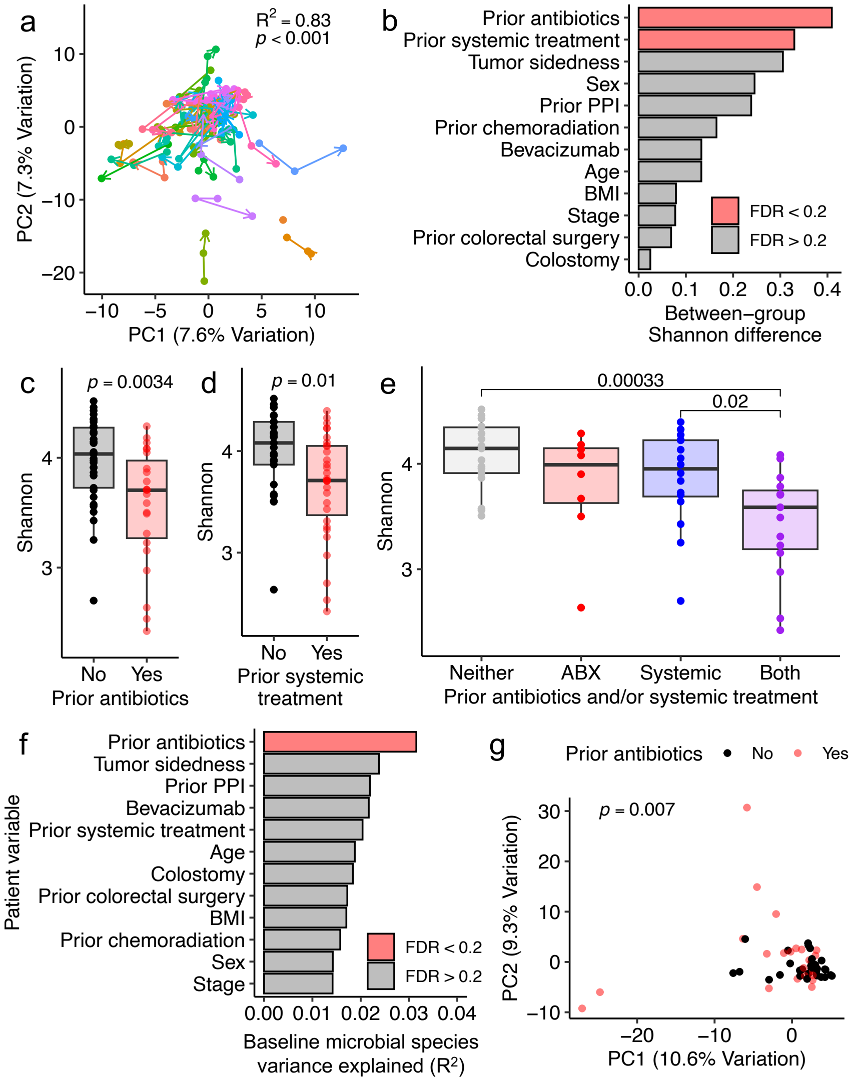

Supplemental Figure 1. Prior antibiotic use and systemic treatment are associated with altered baseline microbial diversity. (a) Species-level principal coordinates analysis (central log ratio (CLR)-transformed Euclidean distances) across all timepoints, colored by patient, with arrows connecting patient samples pointing towards later timepoints (i.e baseline, cycle 3, post). **(b)** Patient demographics and treatment history are associated with bacterial diversity differences (Shannon index) at baseline. **(c-e)** Boxplots of Shannon diversity vs antibiotic use **(c)**, prior systemic treatment **(d)**, or antibiotic use and/or prior systemic treatment (**e**). **(f)** Permutational multivariate analysis of variance (PERMANOVA) testing of patient demographics and treatment history with respect to baseline bacterial taxa composition. **(g)** PCA of CLR-transformed Euclidean distances depicting antibiotic-associated differences in the baseline microbial species. *p*-values: Student's *t* test **(b-e)**, PERMANOVA test with central log ratio (CLR)-Euclidean ordination **(a,f,g).** For **(c-e)**, comparisons with *p*<0.05 are labeled. Benjamini-Hochberg false discovery rate (FDR) correction applied for **(b,f)**, with FDR<0.2 called as significant.

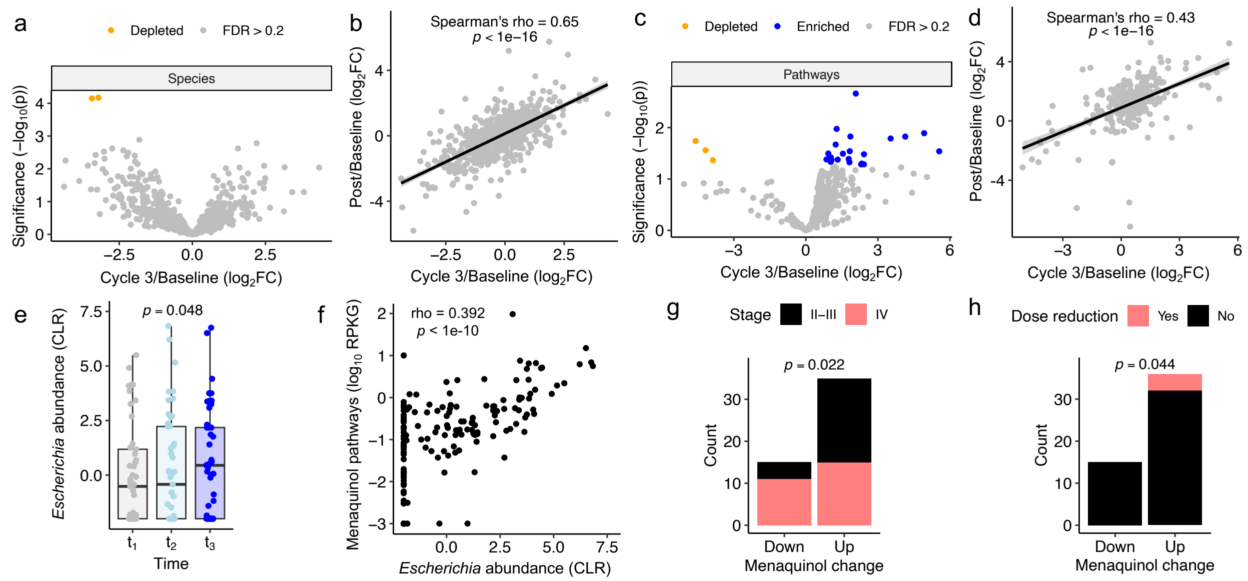

Supplemental Figure 2. **Consistent microbiota shifts during and after treatment. (a)** Volcano plot of differentially abundant microbial species during cycle 3 (t_2) vs baseline (t_1). Orange dots represent significantly depleted species [false discovery rate (FDR)<0.2]. **(b)** Comparison of $\log_2$ fold change of species at cycle 3 (t_2) or post-treatment (t_3) relative to baseline (t_1). **(c)** Volcano plot of pathways during cycle 3 (t_2) vs baseline (t_1). Points represent significantly enriched (blue) and depleted (orange) pathways (FDR<0.2). **(d)** Comparison of $\log_2$ fold change of pathways at cycle 3 (t_2) or post-treatment (t_3) relative to baseline (t_1). **(e)** *Escherichia* abundance vs time. **(f)** *Escherichia* abundance vs menaquinol pathway abundance. **(g,h)** Comparison of menaquinol synthesis gene enrichment during treatment versus cancer stage (**g**) and on-treatment (t_2) dose reduction (**h**). Patients were grouped using average $\log_2$ fold change depicted in **Figure 1h**. *p*-values: Mixed-effects model of abundance vs time, with patient as a random effect (**a,c,e**); Spearman's rank correlation (**b,d,f**); one-sided likelihood-ratio test (**g,h**).

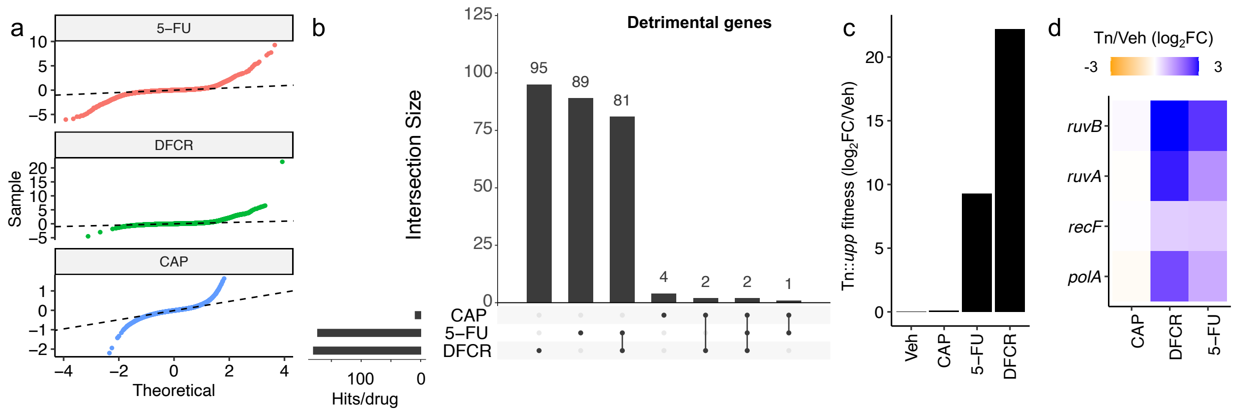

Supplemental Figure 3. Uridine phosphorylase (*upp*) exacerbates fluoropyrimidine toxicity. (a) Quantile-quantile (Q-Q) plot showing deviation from normality (dotted black line) for a RB-TnSeq library treated with 500 µM capecitabine (CAP), 5'deoxy-5-fluorocytidine (DFCR), and 5-fluorouracil (5-FU) relative to Vehicle (Veh). **(b)** Upset plot of significantly enriched transposon-disrupted genes (i.e. the intact gene is detrimental) across all 3 conditions. **(c)** Fitness of Tn::*upp* mutant in all four conditions, relative to vehicle. Values represent the mean of 2 biological replicates. **(d)** Gene set enrichment analysis of detrimental genes from **(b)** revealed homologous recombination as the sole significantly enriched pathway (p<0.01). RB-TnSeq fold changes of detrimental homologous recombination genes are depicted.

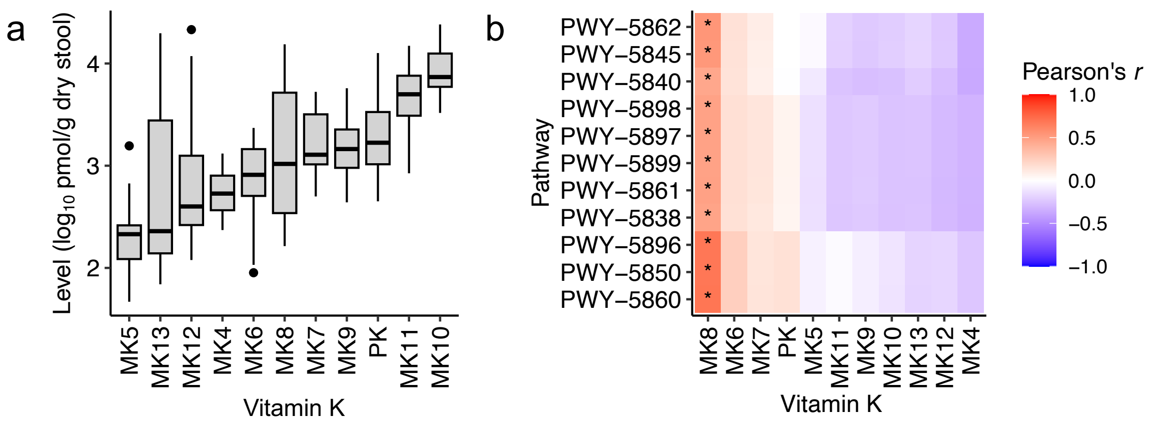

Supplemental Figure 4. Microbial menaquione pathways correlate with stool menaquinone-8 metabolite abundance. (a) Menaquinone and phylloquinone levels measured in stool samples. **(b)** Correlation of menaquinone pathway gene and stool metabolite abundances. *$p<0.05$, Pearson's correlation (r).

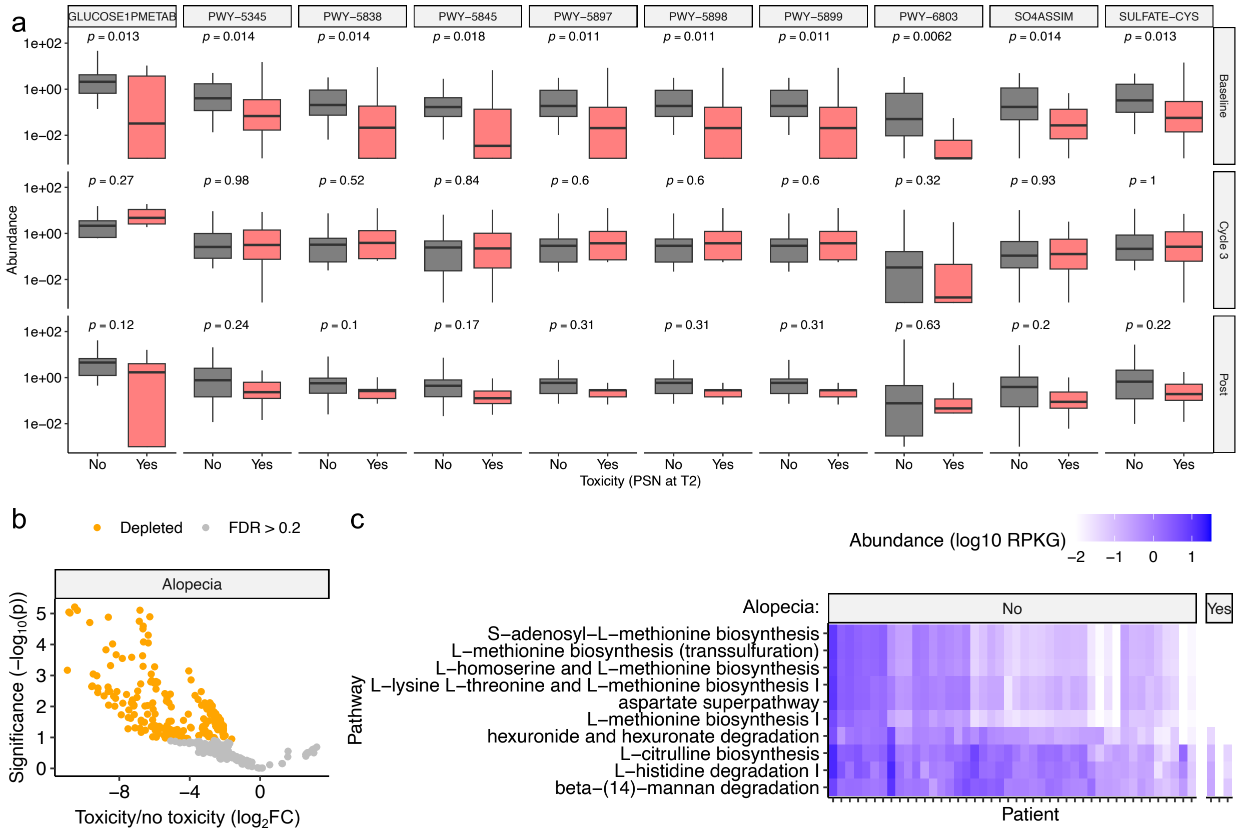

Supplemental Figure 5. Pretreatment microbial gene pathways are associated with development of toxicities during treatment. (a) Abundance of top 10 pathways significantly associated with peripheral sensory neuropathy (PSN) at t_2 (pathways from **Fig. 3d**, labeled with MetaCyc pathway numbers), faceted by pathway and time of stool sample. *p*-values: ANOVA. **(b)** Volcano plot of baseline gene pathways in patients who went on to have alopecia or no alopecia during treatment. Colored points represent significantly depleted (orange) pathways (FDR<0.2). *p*-value: linear model of abundance vs toxicity. **(c)** Heatmap of the baseline (t_1) abundances of the top 10 lowest FDR pathways from **(b)** in units of reads per kilobase per genome equivalent (RPKG), faceted by whether a patient experienced alopecia, with patients and pathways ordered by median hierarchical clustering.

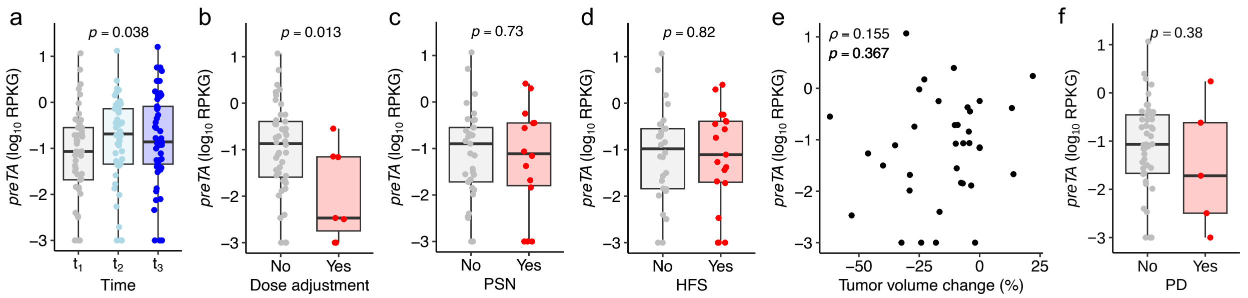

Supplemental Figure 6. Microbial *preTA* increases during capecitabine treatment. (a) *preTA* increases with time. **(b-d)** Baseline *preTA* vs dose delay or reduction **(b)**, peripheral sensory neuropathy (PSN) **(c)**, and hand-foot syndrome (HFS) **(d)**. **(e-f)** *preTA* vs efficacy, as measured by tumor volume change **(e)** or clinical progressive disease (PD) **(f)**. *p*-values: linear mixed-effects model with time as an ordered factor fixed-effect and patient ID as a random effect **(a)**, two-sided Student's *t*-test (**b-d**, **f**), Spearman correlation **(e)**. Number of patients with merged microbiome data and clinical metadata: n=49 **(a)**, 55 **(b)**, 47 **(c)**, 47 **(d)**, 36 **(e)**, and 50 **(f)**.

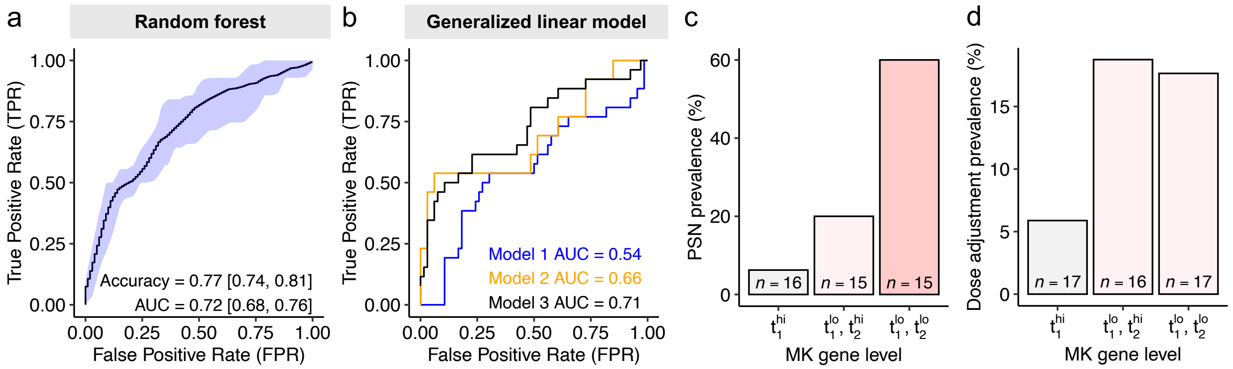

Supplemental Figure 7. Pretreatment microbial gene pathways predict peripheral sensory neuropathy during treatment (at t_2). (a) Receiver operating characteristic (ROC) curve for classification of peripheral sensory neuropathy (PSN; yes/no) with random forest models built with pathways identified in **Fig. 3d**, tested with leave-one-out cross-validation. The black line represents the mean and blue shaded area represents the 95% confidence interval obtained across 100 independent models. Accuracy and area under the curve (AUC) are displayed, with 95% confidence intervals in brackets. **(b)** ROC curves for classification of peripheral sensory neuropathy (yes/no) using 3 generalized linear models, tested with leave-one-out cross-validation. Features used: Model 1 (baseline menaquinone abundance), Model 2 (delta menaquinone abundance), Model 3 (baseline menaquinone abundance, delta menaquinone abundance). Delta abundance was calculated between t_1 and t_2. **(c-d)** Patients were grouped into 3 evenly sized bins based on their menaquinone levels (high t_1 menaquinone, low t_1 menaquinone with relatively higher t_2 menaquinone, and low t_1 and t_2 menaquinone), with group-wise prevalence of t_2 PSN **(c)** or dose adjustment **(d)** displayed. For each patient-time pair in (**a-d**), a single value was used for menaquinone abundance (arithmetic mean of all MetaCyc menaquinone pathway abundances).

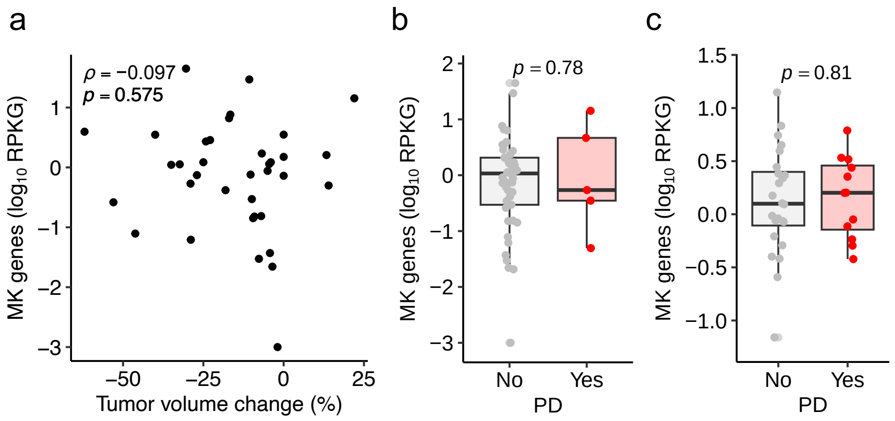

Supplemental Figure 8. Baseline menaquinol gene abundance is not significantly associated with drug efficacy. (a) Tumor volume change vs baseline menaquinol (MK) gene abundance (*n*=36 subjects). **(b-c)** Menaquinol gene abundance in subjects with or without progressive disease (PD) in the Netherlands (**b,** *n*=50) and UCSF (**c,** *n*=36) cohorts. *p*-values: Spearman correlation **(a)**; two-sided Student's *t*-test **(b,c)**. Menaquinol gene abundance calculated as the sum of all KEGG orthologs in **Fig. 1g**.
